# Atlanta Academy of Medicine

**Published:** 1879-01

**Authors:** 


### ATLANTA ACADEMY OF MEDICINE.

ATLANTA, December 2, 1878.

Dr. J. F. Alexander, President, in the chair.

Dr. H. L. Wilson reported a case of anginose scarlet fever. Taken sick on the 23d. On the 24th, headache; had sore throat and sick stomach, with fever and vomiting at night. High fever on the 25th; redness of fauces and enlargement of tonsils. Suspected roseola. Throat causing uneasiness; cauterized it with nitrate silver, and since then has used gargle of alum. On the 26th, eruption well out. Intense itching relieved by inunction of mutton suet and occasional tepid sponging. Nausea was most troublesome. Desquamation going on now. All the typical symptoms well marked.

This was the first case the Doctor had seen since the war. Since the fever has been reduced the pulse has been rather weak, and he has given egg-nogg.

Dr. Salm reported a case, the symptoms in which, as given by patient, were sudden pain over stomach while occupied in store in summer of 1877. Pain continued and extended down left side up and over left shoulder and down right side. In store he has no trouble, but on street is violently attacked. In morning he has attack of belching. About nine years before first taken, had rheumatic fever, and same summer had had rheumatism of foot. He believed it dyspepsia.

Dr. Todd agrees with Dr. Salm in diagnosis. Recommends ingluvin, or if sarcinæ ventriculæ are present, sulphurous acid, or sulphites.

Dr. Calhoun reported a case of enlarged tonsils, producing deafness and difficulty of breathing. The girl, eleven years old, has had the trouble five to six years. Tonsils so much enlarged as to touch. Difficulty of respiration marked at night. Deafness commonly a result of the trouble, produced by chronic inflammation of the eustachian tube, and from pressure on the pharyngeal openings of the tube. Day before yesterday he removed the tonsils. He seized the tonsil with large bulldog forceps, and cut it off with a curved, probe-pointed bistoury; cutting upwards; and found no trouble with the first gland, but the patient struggled when the second was to be removed, but a few whiffs of chloroform quieted her. The child has slept better since the operation than before since the affection began, but cannot tell yet what effect there will be upon her hearing.

He related another case, similar to the one above reported, where hearing improved wonderfully.

Dr. Todd asked the practical difference between the eruption in diphtheria and scarlet fever.

Dr. Wilson does not believe many cases of so-called diphtheria is diphtheria; for in diphtheria eruption is confined to the face, and in scarlatina it is more diffused. In scarlet fever the skin is smooth; in diphtheria it is rough, and is punctate; in scarlatina diffused.

